# Geriatric oncology: comparing health related quality of life in head and neck cancer patients

**DOI:** 10.1186/1758-3284-3-3

**Published:** 2011-01-13

**Authors:** Augusta P Silveira, Joaquim Gonçalves, Teresa Sequeira, Cláudia Ribeiro, Carlos Lopes, Eurico Monteiro, Francisco L Pimentel

**Affiliations:** 1 Oral Anatomy and Oral Histology- Health Sciences Department, ICBAS/UP - Institute for the Biomedical Sciences Abel Salazar ICBAS/UP - Institute for the Biomedical Sciences Abel Salazar ICBAS/UP - Institute for the Biomedical Sciences Abel Salazar Fernando Pessoa University Rua Carlos da Maia, 296, 4200-150 Porto, Portugal; 2 Institute for Biomedical Sciences Abel Salazar- Porto University, Lg. Prof. Abel Salazar no. 2. 4099-003 Porto, Portugal; 3 The Centre of Health Studies and Research of the Coimbra University, Av. Dias da Silva, 165, 3004-512, Coimbra, Portugal; 4 Math Department, Polytechnic Institute of Cávado and Ave, Campus do IPCA - Lugar do Aldão 4750-810 Vila Frescainha S. Martinho Barcelos, Portugal; 5 Institute for Molecular and Cell Biology Rua do Campo Alegre, 823, 4150-180, Porto, Portugal; 6 Health Sciences Department, Portuguese Catholic University, Campus Viseu Estrada da Circunvalação; 3504-505, Viseu, Portugal; 7 Santiago de Compostela University - Facultad De Medicina Y Odontologia, Rua San Francisco, S/N, 15704, Santiago De Compostela, Espanha; 8 Health Sciences Department, Institute for Biomedical Sciences Abel Salazar- Porto University, Lg. Prof. Abel Salazar no. 2. 4099-003 Porto, Portugal; 9 ICBAS/UP - Institute for the Biomedical Sciences Abel Salazar ICBAS/UP - Institute for the Biomedical Sciences Abel Salazar ICBAS/UP - Institute for the Biomedical Sciences Abel Salazar Fernando Pessoa University, Rua Carlos da Maia, 296, 4200-150 Porto, Portugal; 10 Portuguese Institute for Oncology - Porto, Otorhinolaringology service (IPO-Porto, ORL), Rua Dr. António Bernardino de Almeida, 4200-072, Porto, Portugal; 11 Aveiro University, Secção Autónoma Ciências da Saúde; Campus Universitário de Santiago, Aveiro, Portugal; 12 Infante D. Pedro Hospital, Aveiro Av. Artur Ravara, 3814-501, Aveiro, Portugal

## Abstract

**Background:**

Population ageing is increasing the number of people annually diagnosed with cancer worldwide, once most types of tumours are age-dependent. High-quality healthcare in geriatric oncology requires a multimodal approach and should take into account stratified patient outcomes based on factors other than chronological age in order to develop interventions able to optimize oncology care.

This study aims to evaluate the Health Related Quality of Life in head and neck cancer patients and compare the scores in geriatric and younger patients.

**Methods:**

Two hundred and eighty nine head and neck cancer patients from the Oncology Portuguese Institute participated in the Health Related Quality of Life assessment. Two patient groups were considered: the geriatric (≥ 65 years old, n = 115) and the younger (45-60 years old, n= 174). The EORTC QLQ-C30 and EORTC QLQ-H&N35 questionnaires were used.

**Results:**

Head and neck cancer patients were mostly males, 77.4% within geriatric group and 91.4% among younger patients group.

The most frequent tumour locations were similar in both groups: larynx, oral cavity and oropharynx - base of the tongue.

At the time of diagnosis, most of younger male patients were at disease stage III/IV (55.9%) whereas the majority of younger female patients were at disease stage I/II (83.4%). The geriatric patient distribution was found to be similar in any of the four disease stages and no gender differences were observed.

We found that age (geriatrics scored generally worse), gender (females scored generally worse), and tumour site (larynx tumours denounce more significant problems between age groups) clearly influences Health Related Quality of Life perceptions.

**Conclusions:**

Geriatric oncology assessments signalize age-independent indicators that might guide oncologic geriatric care optimization. Decision-making in geriatric oncology must be based on tumour characteristics and chronological age but also on performance status evaluation, co-morbidity, and patient reported outcomes assessment.

## Background

The United Nations Organization (UNO) considers the period between 1975 and 2025 the ageing era, once society is ageing and life expectancy is rapidly growing. This progressive ageing of the worldwide population is increasing the number of people annually diagnosed with cancer once most types of tumours are age-dependent. In Europe, sixty percent of new cancer cases and over seventy percent of cancer deaths occur in patients aged 65 years and older [1,2].

Head and neck cancer is one of the sixth most prevalent worldwide neoplasm, with an estimated 900,000 new cases diagnosed annually. Independently of tumour site (oral cavity, oropharynx, sinus and nose, salivary glands, larynx), surviving patients experience a deterioration of their basic functions affecting such important functions as: breathing, mastication, salivating, swallowing, speaking, senses (hearing, taste and smell). Moreover, aesthetics appearance is perceived as profoundly affecting their lives [3-7].

High-quality healthcare in geriatric oncology (GO) requires a multimodal approach. The stratified patient outcome based on factors other than chronological age supports development of more effective interventions in order to optimize oncology care.

Geriatric patients are often under-treated, largely under-represented in cancer trials and do not receive standard treatments because they are considered unfit for treatment as a consequence of inaccurate estimation of the operative risk [8-11].

Health Related Quality of Life (HRQoL) begun to be considered as one of the hard end-points for clinical and research in GO. Such assessment can promote better patients selection and support treatment decisions. It may provide new measures and interventions in order to optimize the individual treatment plan and reducing inappropriate age-related inequity found in healthcare assessment [10,12,13].

The HRQoL assessment involves biological, psychological and socio-cultural criteria in a multidimensional perspective looking for well-being ageing indicators: longevity, biological, social and mental health, social competence and status, satisfaction, cognitive control and efficiency, productivity, activity, income, family and occupational roles and informal relations continuity. Methodological problems elderly related - illiteracy, concomitant diseases, social disorders, unviable validated instruments or trouble in question understanding - make the assessment a true challenge [14,15].

HRQoL directed programs for GO professionals - developed in a multidisciplinary approach and focused on advanced research and clinical practice - could optimize HRQoL of both patients and their relatives.

Indeed, essential factors are recognized as important to expedite the progress and healthcare systems refining: the need of dedicated investigators, continuous health education planning and linkage efforts among comparative and multidisciplinary groups [16-18].

This study aims to evaluate the HRQoL in head and neck cancer patients (HNCP) and compare the scores in geriatric and younger patients.

## Methods

### Ethics

The study was carried out in compliance with the Helsinki Declaration. The methodology was previously approved by the local research ethical committee and all HNCP agreed to participate in the research and gave their informed consent. The data were collected for research purposes as part of the routine evaluation.

### Patients

From January 2010 to July 2010, consecutive outpatients - 3-9 months after first treatment completion- admitted to the Otorhinolaringology and Head and Neck Services (ORL and H&N service) IPO-Porto, Portugal, were invited to participate in the HRQoL assessment protocol of ORL and H&N Services. 289 patients were assessed and questionnaires were completed immediately before consultation as a part of the routine evaluation. Inclusion criteria were ability to understand written and spoken Portuguese and provision of written consent.

According to literature 65 years old is frequently considered a significant mark along the ageing process commonly overlapping retirement. In order to understand the influence of age in HRQoL patients were divided into two groups: the geriatric patients - GP (≥ 65 years old, n = 115) and younger patients - YP (45-60 years old, n = 174).

### Socio-demographic and clinical data

Clinical data - such as tumour diagnosis and location, tumour staging, tobacco habits, feeding type and presence of traqueostomy - as well socio-demographic data - age and gender - were collected from the patient's clinical process and complemented, when needed, in semi structured interviews.

### Questionnaires

Quality of Life of HNCP was assessed using the European Organization for Research and Treatment of Cancer Quality of Life Questionnaire-Core 30 (EORTC QLQ-C30) and the European Organization for Research and Treatment of Cancer Quality of Life Questionnaire Head and Neck Cancer Module (EORTC QLQ-H&N35) - Portuguese version.

The EORTC QLQ-C30 (version 3.0) is a questionnaire developed to assess the HRQoL of cancer patients. It consists of 30 questions: twenty four form nine multi-item scales presenting various aspects of HRQoL: five functional scales (PF, Physical functioning; SF, Social functioning; EF, Emotional functioning; RF, Role functioning; CF, Cognitive functioning), three symptom scales (fatigue, pain, nausea and vomiting) and a global condition (health and quality of life). The remaining six are single-item scales describing different cancer relevant symptoms. During the scoring procedure, raw EORTC QLQ-C30 scores are linearly transformed into 0 e100 scales. For global health status and the five functioning scales, a score of 100 corresponds to a high HRQoL. For financial difficulties and the eight symptoms, a score of 100 implies maximum difficulty or symptom burden. The additional module - QLQ-H&N35 (version 3.0) - is disease-specific for head and neck patients. It consists of 35 questions organized in seven symptoms multi-item scales (twenty four questions are presented) and eleven are single-item scales describing different specific concerns of these head and neck cancer patients.

### Analysis Strategies and Statistics

Completed questionnaires were scored according to the developers' instructions.

HRQoL data were analyzed by the Statistical Package for Social Sciences (SPSS), version 17 for windows. Descriptive data are presented with means, standard deviations, medians, ranges, and proportions as appropriate.

## Results

### Patients Characteristics

Male HNCP constituted the majority in the overall (77.4%), particularly in the YP group where they represented 91.4% (Table 1).

**Table 1:** Socio-demographic characteristics of HNCP (Age/Gender).

Age groups n = 284		Gender - Number of subjects (%)							
		
		40-60		≥ 65		Male		Female	
40-60	≥ 65	Male	Female	Male	Female	40-60	≥ 65	40-60	≥ 65
174(59.5%)	115(40.5%)	159(91.4)	15(8.6)	89(77.4)	26(22.6)	159(64.1)	89(35.9)	15(36.6)	26(63.4)

The most frequent tumour locations were the larynx, oral cavity and oropharynx - base of the tongue in both groups: 29.6%, 12.6%, and 12.6% for GP and 31.4%, 16.2% and 10.6% for YP respectively.

At the time of diagnosis, most YP males were at disease stage III/IV (55.9%) whereas the majority of YP females were at disease stage I/II (83.4%). YP group were treated with surgery (71.4%), radiotherapy-RT (7.6%), chemotherapy-CT (9.7%), and RT + QT (11.3%); the GP were treated with surgery (79.1%), RT (14.3%), CT (2%), and RT + QT (4.6%).

**Figure 1:**
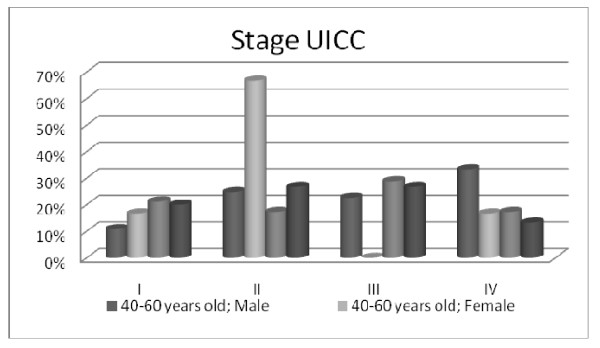
**Socio-demographic and clinical characteristics of HNCP (Age/Gender/Tumour Staging)**.

**Figure 2:**
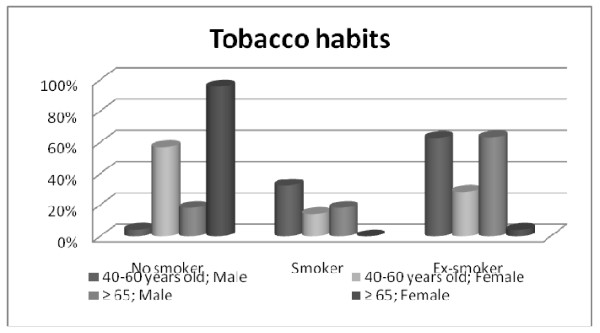
**Socio-demographic and clinical characteristics of HNCP (Age/Gender/Tobacco Habits)**.

The GP gender distribution along disease stages disease was uniform. The tobacco habits inquiry revealed that males are predominantly ex-smokers and females are mostly non smokers being this tendency more evident in geriatric female patients (Figures 1 and 2).

Most HNCP were normally feed in both groups but when considering non-oral feeding, PEG tube feeding was always the major choice particularly found in the GP group (Table 2).

**Table 2:** Socio-demographic and clinical characteristics of HNCP (Age/Feeding/Traqueostomy).

Age	Feeding No. of subjects (%)			Traqueostomy No. of subjects (%)	
	
	Oral feeding	PEG tube	Nasogastric tube	Yes	No
**40-60**	154 (88.5)	8 (10.6)	4(1.0)	23 (21.9)	82 (78.1)
**≥ 65**	92 (92.8)	11(4.8)	1(2.4)	38 (23.3)	125 (76.7)

### EORTC QLQ-C30 and QLQ-H&N35

EORTC QLQ-C30 data are shown in Table 3. The geriatric HNCP scored worse compared with the younger sample on the functional scales: Physical, Cognitive and Emotional. The differences obtained in Physical and Emotional functioning were both clinically (difference ≥10 points) and statistically significant.

**Table 3:** Results from the EORTC QLQ-C30 for subgroups of HNCP (Gender/Age).

EORTC QLQ C30		Male		Female	
		
		40-60 (n = 159) % (SD)	≥ 65 (n = 89) % (SD)	40-60 (n = 15) % (SD)	≥ 65 (n = 26) % (SD)
**Functional Scales**	Physical functioning	79 (18)	72 (25)	83 (19)	68 (23)
	Role functioning	82 (26)	79 (30)	78 (30)	79 (26)
	Cognitive functioning	85 (22)	79 (23)	79 (28)	78 (24)
	Emotional functioning	78 (21)	75 (26)	62 (32)	69 (23)
	Social functioning	85 (23)	85 (21)	82 (32)	85 (22)
**Single Items**	Dyspnoea	17(28)	22 (29)	9 (27)	10 (18)
	Insomnia	25 (30)	31 (37)	29 (33)	20 (27)
	Appetite loss	18 (29)	22 (33)	24 (37)	21 (31)
	Constipation	10 (24)	21 (32)	13 (28)	14 (25)
	Diarrhoea	6 (15)	9 (24)	0	3 (13)
	Financial Difficulties	29 (35)	14 (25)	22 (35)	14 (21)
**Symptoms Scales**	Fatigue	27 (24)	29 (24)	27 (28)	26 (21)
	Nausea and vomiting	6 (14)	4 (10)	17 (28)	5 (12)
	Pain	25 (28)	25 (30)	26 (26)	23 (23)
**Global Health Status/QoL**		59 (26)	59 (22)	59 (25)	54 (23)

Male GP presented a worse score in all single items and symptoms scales examined - dyspnoea (22), insomnia (31), constipation (21), diarrhoea (9), and fatigue (29). On the contrary, financial difficulties (29), appetite loss (24), and nausea and vomiting (17) caused a greater negative impact in younger HNCP (40-60 years old), mainly in females HNCP. The Standard Deviation (SD) values show that the sample is very heterogeneous in all the parameters evaluated.

Similar outcomes were observed for Global Health Status/QoL in all subpopulations, around 58 points, with lower scores found in geriatric females HNCP (54).

QLQ-H&N35 data are shown in Table 4. The female HNCP aged 40-60 years old scored considerably worse in most domains. The exceptions focused on Swallowing (23), and Sticky saliva (37) that obtained inferior scores in geriatric males, Coughing (29) with poor results in males aged 40-60 years old and Dry Mouth (48) with lower outcomes in geriatric females.

**Table 4:** Results from the EORTC QLQ-H&N35 for subgroups of HNCP (Gender and Age).

EORTC QLQ H&N 35		Male		Female	
		
		40-60 (n = 159) % (SD)	≥ 65 (n = 89) % (SD)	40-60 (n = 15) % (SD)	≥ 65 (n = 26) % (SD)
**Symptoms Scales**	Pain	22 (22)	21 (25)	26 (21)	20 (18)
	Swallowing	22 (28)	23 (28)	16 (20)	19 (25)
	Senses Problems	20 (28)	20 (30)	22 (30)	20 (29)
	Speech Problems	27 (25)	29 (28)	35 (37)	18 (19)
	Trouble With Social Eating	19 (24)	24 (29)	22 (24)	19 (26)
	Trouble With Social Contact	14 (17)	15 (21)	17 (24)	11 (16)
	Less Sexuality	26 (32)	39 (34)	40 (39)	31 (40)
**Single Items**	Teeth	27 (36)	33 (40)	33 (40)	27 (35)
	Opening Mouth	22 (30)	17 (30)	22 (27)	18 (25)
	Dry Mouth	42 (35)	42 (36)	36 (55)	47 (37)
	Sticky Saliva	33 (33)	37 (35)	29 (33)	33 (39)
	Coughing	29 (29)	25 (23)	13 (21)	14 (23)
	Felt Ill	20 (29)	16 (26)	24 (32)	20 (29)
	Pain killer	15 (17)	15 (17)	22 (1)	19 (17)
	Nutritional Supplements	4 (10)	4 (11)	9 (15)	3 (9)
	Feeding Tube	5 (12)	4 (11)	0	3 (9)
	Weight Loss	11 (16)	11 (16)	11 (16)	11 (16)
	Weight Gain	8 (15)	6 (12)	11 (16)	3(9)

### Tumour location

When EORTC QLQ-C30 scores are compared according to tumour location and age, it is verified that in Larynx tumours, GP reveal worse values. For all subpopulations considered, Physical (70), and Role functioning (75), Constipation (22) and Fatigue (33) revealed the lowest values. Oral Cavity analysis demonstrates that younger HNCP had poor results in Social Functioning (80), Diarrhoea (10) and Financial Difficulties (30). HNCP aged 40-60 years old with Pharynx (base of the tongue) cancer reveal a negative impact in HRQoL in several domains in opposition to the GP that appointed Physical (71) and Cognitive Functioning (78) as the worst domains.

The EORTC QLQ-H&N 35 scores identified GP with Larynx cancer as expressing poor score in all HRQoL domains with the exceptions of Dry Mouth and Felling Ill. The differences between geriatric and YP were found significant in the Swallowing (29 to 17), Speech (34 to 42), Eating (16 to 28), Social Contact (13 to 21), Sexuality (17 to 44) Scales, and Teeth Problems (17 to 33). Considering Oral cancer, no significant differences were found between these groups, except the single item: Teeth Problems that scored worse in geriatric HNCP (15 to 33). When Pharynx (base of the tongue) cancer is considered, there are not significant differences between older and younger cancer patients, with scores lightly better in geriatric cancer patients. The exceptions focused on Speech (34 to 21), Open Mouth (28 to 18), Laughing (40 to 11), and Felt Ill (27 to 13), were found to be quite inferior in patients aged 40-60 years old.

## Discussion

Head and neck cancer occurs mainly between the fifth and sixth age decade, being the number of elderly HNC rising as the result of demographic changes. Aged patients are predisposed to spontaneous mutations and hypomethylation of the DNA, important predisposition factors for tumour cell transformation and oncogen activation and thus favouring tumour development. Actually, it is observed an increase of head and neck cancer in geriatric population [9,19].

The participants, all from North of Portugal, are mainly males (86%) and mostly young (64%). Such gender asymmetry seems to be associated with cumulative risk factors exposure (oral hygiene, dental status, oral mucosal lesions, alcohol and tobacco use, virus infection and lifestyle). Moreover, the spontaneous mutations aged-related may have different expression in males and females [20,21].

Considering tumour location, our data are in agreement with literature identifying oral cavity, oropharynx, hypopharynx, and larynx as the most common sites for head and neck cancer [22]. Furthermore, the more prevalent stages found were III and IV in males and II and III in females. Age seems not interfere in this tendency.

The male participants are predominantly ex-smokers and smokers and female HNCP are mainly non smokers. This corroborates other findings showing that men are more likely than women to be current smokers [23-26].

Head and neck cancer is an extremely distressing disease disturbing anatomy and physiology of cervicofacial region and affecting important functions - vision, hearing, balance, olfaction, taste, mastication, swallowing, breathing, voice - endocrine balance and body image. Consequently, when considering HNCP, the impact of the diagnosis and treatment on the multidimensional patient outcomes need the most serious consideration. Accordingly, HRQoL assessment in head and neck cancer is essential to know the HNCP perceptions.

Derks (2004) evaluated seventy-eight older (> or = 70 years) and 105 younger HNCP (45-60 years) and referred that before and after treatment, the physical functioning of the older was worse than that of YP [27]. Fang (2010) described that pretreatment HRQoL variables from patients with nasopharyngeal carcinoma, provided available prognostic value for distant metastasis and survival, particularly physical functioning. Additionally, other studies support the correlation of patient reported HRQoL scales with survival in head and neck cancer [28,29].

Infante-Cossio reported in 2009 that HRQoL seemed to be associated with age, indicating that HNCP under 65 years scored better [30]. Our results corroborate these findings demonstrating that geriatric HNCP globally scored worse. The YP revealed more problems related to Financial Difficulties, Appetite Loss, and Nausea and Vomiting.

Results from the QLQ-H&N35 revealed YP female scored considerably worse in the majority of the domains - these findings are similar with those described in literature identifying advanced tumour stage, female gender, and long-term follow-up as the factors that adversely affect HRQoL in HNCP [30,31].

The Global Health Status/QoL remains generally comparable between groups and according to van der Schroeff (2007) who found no significant differences between older and younger HNCP when considering survival or overall HRQoL [32].

HRQoL scores revealed to be associated with tumour location and age: Larynx tumours reveal more problems in GP when considering Physical, Role functioning, Constipation and Fatigue. Oral Cavity analysis indicated that younger HNCP had poor scores in Social Functioning, Diarrhoea and Financial Difficulties. Unexpectedly, YP with Pharynx cancer (base of the tongue) revealed a negative HRQoL impact in several domains - the opposite of GP that scored better in almost all domains.

The EORTC QLQ-H&N 35 scores identified that GP with Larynx cancer have poor results in all HRQoL domains. In Oral and Pharynx cancers (base of the tongue) there are no significant differences between the considered groups, being the scores lightly better in GP.

Presently, significant uncertainty exists in GO. The GP often do not receive aggressive therapy regimens as a consequence of imprecise evaluation of the operative risk. A comprehensive geriatric assessment and team approach may contribute to the identification of many previously unmet problems, allowing then better strategies planning in order to improve health outcomes [33].

There is almost an international consensus that HNCP should be treated with curative intention and aggressive treatment option should not be excluded [34,35]. Geriatric HNCP should be considered to receive standard treatment - in fact previous studies reveal that therapy associated complications do not occur significantly more often in these patients. Age alone should not be the basis for therapeutic planning and decision-making in geriatric HNCP [9,36,37].

HRQoL assessment has been introduced as one of the hard end-points in GO. Survival remains the most significant end point and a HRQOL evaluation should be thus a secondary end point. In fact, an EORTC questionnaire module for older people with cancer was recently developed, the EORTC QLQ-ELD15. Such questionnaire is prepared for large-scale studies in combination with EORTC QLQ-C30 [38,39].

HRQoL assessment should be integrated in the comprehensive geriatric assessment procedure in order to meet the GO healthcare professional's improvement expectations [8,18,39].

## Conclusions

Worldwide oncology programs have been developed for the individualized management of HNCP in a multidimensional and multidisciplinary way.

This study reveals that geriatric HNCP globally scored worse in HRQoL assessment. Additionally advanced tumour stage, female gender, and long-term follow-up are variables that adversely affect HRQoL in HNCP.

Therapeutic planning and decision-making in Head and Neck Cancer must be based not only on tumour characteristics and chronological age, but also on performance status evaluation, co-morbidity and patient reported outcomes assessment. These outcomes associated with comprehensive HNCP assessment aims to screen unmet problems, contributing as prognosis predictors, substantiating the decision process, and reducing age-related inequity. These assessments are signalizing age-independent indicators that do optimize oncologic geriatric care.

## Competing interests

The authors declare that they have no competing interests.

## Authors' contributions

AS conceived of study, participated in the acquisition, analysis and interpretation of data, drafted and submitted the manuscript. JG conceived of the study, participated in the acquisition, analysis and interpretation of data, and performed the statistical analysis. TS conceived of study, participated in the acquisition, analysis and interpretation of data, helped to draft the manuscript and revised it critically. CR participated in the analysis and interpretation of data, and revised the manuscript. CL, EM and FLP conceived of the study, participated in its design and coordination, helped to draft the manuscript, and revised it critically for important intellectual content. All authors read and approved the final manuscript.
